# DNA methylation of the *p14^ARF^*, *RASSF1A* and *APC1A* genes as an independent prognostic factor in colorectal cancer patients

**DOI:** 10.3892/ijo.2012.1682

**Published:** 2012-10-30

**Authors:** TORBJÖRN K. NILSSON, ZARAH M. LÖF-ÖHLIN, XIAO-FENG SUN

**Affiliations:** 1 Department of Laboratory Medicine, Örebro University Hospital;; 2 School of Health and Medical Sciences, Örebro University, Örebro;; 3 Department of Oncology, Institute of Clinical and Experimental Medicine, Faculty of Health Sciences, University of Linköping, Linköping, Sweden

**Keywords:** *APC1A*, colorectal cancer, CpG sites, DNA methylation, *O^6^-MGMT*, *p14^ARF^*, *p16^INK4a^*, preterapeutic predictor, prognosis, Pyrosequencing^®^, *RASSF1A*, survival, tumor stage, tumor differentiation

## Abstract

We quantitated the methylated fraction of CpG sites in the promoter regions of *O^6^-MGMT*, *p14^ARF^*, *p16^INK4a^*, *RASSF1A* and *APC1A* in tumor tissue from patients with colorectal cancer (CRC) in order to determine if promoter hypermethylation of any of these genes predicts survival. DNA was isolated from 111 primary CRC and 46 matched normal colorectal mucosa samples from the same patients, obtained at primary surgery and DNA methylation was examined by Pyrosequencing^®^. Follow-up time was up to 20 years. Patients showed partial promoter methylation in the following frequencies: *O^6^-MGMT*, 34%; *p14^ARF^*, 29%; *p16^INK4a^*, 28%; *RASSF1A*, 14%; and *APC1A*, 27%. Normal mucosa was always unmethylated. CRC patients with methylated *p14^ARF^* gene promoter had significantly worse prognosis (p=0.036), whereas those with methylated *O^6^-MGMT* had significantly better prognosis through the first 60 months post-treatment (RR 0.36; p=0.023). Methylation of one or more of the genes from the set *p14^ARF^*, *RASSF1A* and *APC1A*, was significantly (p= 0.021) associated with worse prognosis even adjusting for tumor stage and differentiation (RR 2.2, p=0.037). Thus, DNA methylation of the *p14^ARF^*, *RASSF1A* and *APC1A* genes, diagnosed by Pyrosequencing, defines a poor prognosis subset of CRC patients independently of both tumor stage and differentiation. *O^6^-MGMT* methylation may play a protective role.

## Introduction

DNA methylation is a common feature often seen in tumor suppressor and DNA repair genes (1). Methylation of CpG sites in the promoters of these genes frequently causes loss of expression, affecting cell cycle regulation, cell adhesion or DNA reparation. Several genes have been reported to be involved, through DNA methylation, in sporadic colorectal cancer (CRC) (2,3). Here, we focus on five of these, *O^6^-MGMT*, *p14^ARF^*, *p16^INK4a^*, *RASSF1A* and *APC1A*(4–9). Studies on the methylation status of the promoter regions of these genes in CRC development reported frequent promoter hypermethylation in tumor tissue DNA, whereas normal tissue DNA remained unmethylated (10,11).

Methylation specific PCR, a common technique to study DNA methylation, assays the methylation status of a few CpG sites (i.e., those interfering with the PCR primer binding) and only gives a qualitative indication (methylated or not). Like all ‘allele specific’ PCR methods, it depends largely on a combination of the number of PCR optimization experiments and individual judgment of presence or absence of bands. We do not know which particular CpG sites will silence these suppressor genes when methylated because the standard methods assay only a few of the sometimes 100 or so CpG sites (12). These methodological concerns stall our understanding of the clinical role of DNA methylation.

We have therefore developed Pyrosequencing^®^ assays (13) to get a DNA sequence-specific as well as quantitative measure of DNA methylation of the promoter CpG sites as well as the adjacent CpG sites, of the DNA repair gene *O^6^-MGMT* and the 4 tumor suppressor genes *p14^ARF^*, *p16^INK4a^*, *RASSF1A* and *APC1A* previously studied by MS-PCR (10,11). We report our data on tumor biopsies from subjects diagnosed with colorectal cancer or adenomas and adjacent normal mucosa, and furthermore we assessed if these assays had any long-term prognostic value.

## Materials and methods

### Subjects

The study included 111 randomly selected patients with primary colorectal adenocarcinoma who underwent surgical resection at Linköping Hospital, Linköping and Vrinnevi Hospital, Norköping, Sweden. In 46 of the patients, normal mucosa specimens taken from the margin of the resected tumor were also available. The study also included 10 patients with colorectal adenomas, from 7 of whom matched normal mucosa specimens were also available. The patients’ gender, age, tumor location and stage were obtained from surgical and/or pathological records at Linköping and Vrinnevi Hospitals. Tumor differentiation was graded into well moderately or poorly differentiated. The study was approved by the Regional Ethics Review Board in Linköping and an informed consent document was signed by participants.

### DNA isolation and bisulfite treatment of tissue DNA

Genomic DNA was isolated from 20 mg of colorectal tumor tissue (n=111) by means of the Wizard^®^ SV Genomic DNA Purification System according to the manufacturer’s instructions (Promega, Madison, WI, USA). For some of these patients (n=46) distant normal colorectal mucosa tissue was also available and DNA was isolated in the same way. Genomic DNA was also isolated from 20 mg of colorectal adenoma tissue (n=10 subjects), and for several of these subjects (n=7) also from 20 mg of distant normal colorectal mucosa, by means of the QIAamp DNA Mini Kit according to the manufacturer’s instructions (Qiagen Inc., Valencia, CA, USA).

Approximately 1,000 ng of isolated and precipitated DNA was used for the bisulfite treatment. The bisulfite treatment was performed with EZ DNA Methylation kit according to the instructions by the manufacturer (Zymo Research, Orange, CA, USA) except that the incubation time was shortened to 10 h. In short, DNA was diluted with M-Dilution buffer and was incubated for 15 min at 37°C. After the incubation CT conversion reagent was added and the samples were incubated at 50°C for 10 h. The samples were then incubated on ice for 10 min and then M-Binding buffer was added. The samples were centrifuged and then washed using centrifugation and M-Wash buffer. The bisulfite treated DNA was eluted in 10 *μ*l M-Elution buffer and then diluted 4 times with TE buffer (10 mmol/l Tris-HCl, 0.05 mmol/l EDTA, pH 7.5).

### PCR and Pyrosequencing

PCR and Pyrosequencing of the promoter regions of the *O^6^-MGMT*, *p14^ARF^*, *p16^INK4a^*, *RASSF1A* and *APC1A* genes was performed as previously described (13). Pyrosequencing technology was used to sequence-specifically quantitate each of the CpG sites, which are analyzed in practice as C/T-polymorphisms, where a 100% C-reading denotes a fully methylated C (^Me^C=100%) in the original gDNA sample whereas a 100% T-reading denotes that this locus was unmethylated (^Me^C=0%) in the original gDNA. Intermediate ^Me^C percentages denote partial methylation at the level of the sample. Partial methylation, when present, is presumed to be partly owing to admixture of unmethylated non-neoplastic cell types present in the tissues to a varying extent.

### Statistical analysis

The significance of the difference of promoter region hypermethylation on the *O^6^-MGMT*, *p14^ARF^*, *p16^INK4a^*, *RASSF1A* and *APC1A* genes in between normal mucosa samples and primary tumors was tested by χ^2^ or McNemar’s method. The relationships between the promoter region hypermethylation and other factors were examined by the χ^2^ test. The relationship between the expression and survival was tested using Cox’s Proportional Hazard Model. Survival curves were calculated using the Kaplan-Meier method. Two-sided p-values of <0.05 were considered as statistically significant.

## Results

### Prevalence and extent of DNA hypermethylation

Samples from 111 colorectal cancer tumors, 10 colorectal adenomas and 53 matched normal tissues from the same patients (46 from CRC patients, 7 from adenoma patients) were analyzed. Assay failure rate was 0% for all 5 genes studied. All the 53 available paired normal tissues, both from tumor and adenoma tissues were found to be 100% unmethylated on the promoter regions of all the genes. The pyrograms of many of the tumor samples, on the other hand, showed methylation peaks, amounting to a consistent but individual-specific mean percentage of methylated fraction of gene promoter CpG sites. In Table I the number of patients with methylation-positive tumors or adenomas, as well as their mean methylated fractions (%^Me^C) of CpG sites, are shown. Promoter hypermethylation in the CRC samples was commonest for the *O^6^-MGMT* gene (34% of the patients) and least frequent for *RASSF1A* (14%). Adenomas showed a similar pattern.

**Figure 1.**
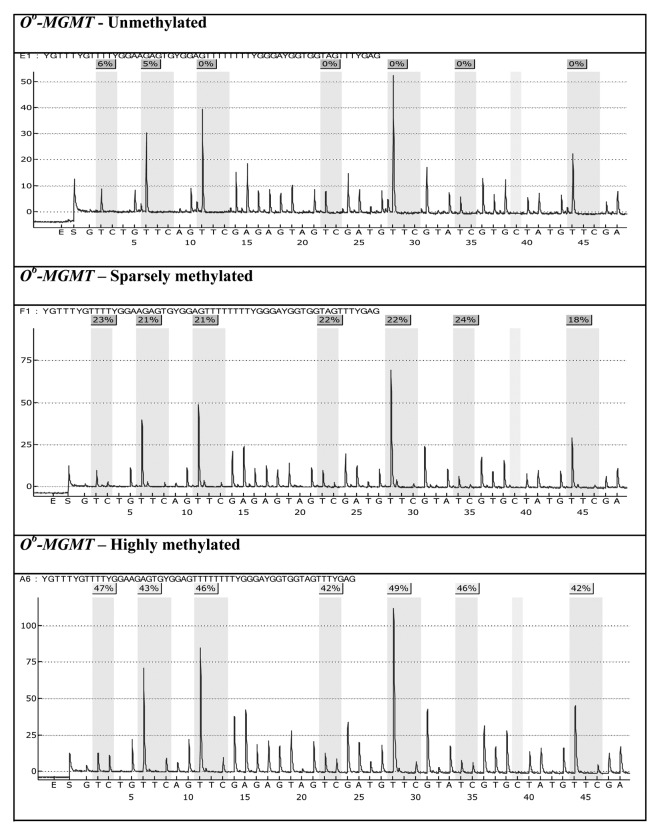
Typical pyrograms of the *O^6^-MGMT* gene showing normal colon mucosa tissue with unmethylated CpG sites (upper panel, %^Me^C <10), CRC tissue with sparsely methylated CpG sites (middle panel, %^Me^C = 18–23) and CRC tissue with highly methylated CpG sites (lower panel, %^Me^C = 42–47).

**Figure 2.**
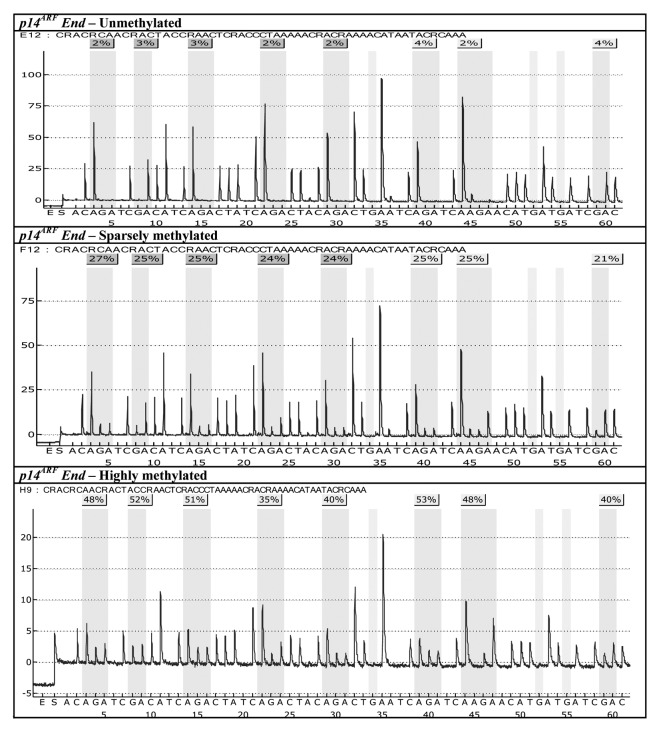
Typical pyrograms of the *p14^ARF^* gene showing normal colon mucosa tissue with unmethylated CpG sites (upper panel, %^Me^C <10), CRC tissue with sparsely methylated CpG sites (middle panel, %^Me^C = 21–27) and CRC tissue with highly methylated CpG sites (lower panel, %^Me^C = 35–53).

The methylation pattern throughout the promoter regions of all the genes was consistent within each individual patient: if methylation was detected for a sample all the CpG sites in the entire promoter region of that gene were methylated at roughly the same proportion (%^Me^C, as obtained from the Pyrograms). Representative patients showing non-methylated normal mucosa and tumor tissues methylated to varying degrees are displayed for *O^6^-MGMT* and *p14^ARF^* in Figs. 1 and 2. While the occurrence of methylated promoter CpG sites varied (Table I), within each patient the methylated fraction (%^Me^C) varied very little between the different CpG sites of a particular gene. The mean methylated fractions stated in Table I are the inter-individual (total-sample) mean values of %^Me^C of CpG sites though the entire promoter regions of all methylation-positive tumor samples.

Concurrent methylation of two or more genes in the colorectal cancer tumors are summarized in Table II. We found 42 patients (38%) to be methylated on one gene, 18 (16%) were methylated on two genes, 15 (14%) on three genes, 5 (4.5%) were found to be methylated on four genes and 1 (0.9%) was methylated on all the genes studied. Out of the 111 tumors, 30 (27%) were thus found to be unmethylated on all genes.

### DNA hypermethylation and long-term outcome

Survival plots for patients with or without promoter methylation for each of the five genes were analysed. In univariate analysis two genes reached statistical significance. Hypermethylation of *p14^ARF^* was related to worse survival (p=0.036) but its significance was attenuated in multivariate analysis when adjusting for tumor stage and differentiation (p=0.065). Hypermethylation of *O^6^-MGMT* was associated with better survival through the first 60 months of follow-up, the risk ratio was 0.36 (95% CI 0.15–0.87, p= 0.049) and still remained significant after adjusting for tumor stage and differentiation (p=0.023), see Table III.

**Figure 3.**
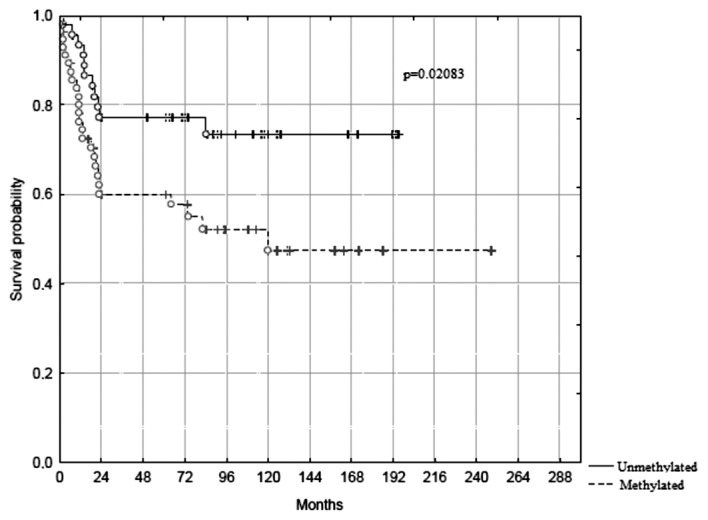
Survival plot of subject with or without methylation on one or more of the genes *p14^ARF^*, *RASSF1A* and *APC1A*.

Since promoter hypermethylation of *p14^ARF^*, *RASSF1A* and *APC1A* all were associated with a similar tendency towards worse prognosis, and many patients were methylated on more than one gene (Table II), we examined whether methylation of any of the three genes (i.e., one or more), would improve prediction of survival. Indeed, hypermethylation of one or more of these three genes defined a set of patient with a significantly (p=0.021) worse long-term survival (Fig. 3), where only ∼45% were still alive in the methylated group by 20 years of follow-up, compared to ∼75% in the unmethylated group. Adjusting for tumor stage and differentiation did not attenuate this association (risk ratio 2.20; 95% CI, 1.05–4.62, p=0.037; Table IV). No association could be found between promoter hypermethylation of these three genes and the other clinicopathological factors including gender, age, tumor location, or tumor stage and differentiation (p>0.05 for all variables).

## Discussion

Aging and environmental factors may lead to neoplasia and cancer. This process usually involves changed expression pattern of genes involved in adhesion, proliferation, differentiation, cell growth, migration and apoptosis, and can be due to mutations, genetic rearrangements, chromosomal instability or promoter hypermethylation (14). In this study we have focused on five such genes, previously suggested to be involved in the development of CRC; *O^6^-MGMT*, *p14^ARF^*, *p16^INK4a^*, *RASSF1A* and *APC1A*(3–12). Methylation of the promoter regions of some of these genes in tissues or serum from patients with CRC has been reported (15–25), but data on the long-term prognostic implications of the whole set is limited.

By using Pyrosequencing, a technique that offers a unique opportunity to quantitate, site-specifically, the methylated fraction in partially methylated CpG sites, we demonstrated that the promoter regions of one or more of the genes analyzed are methylated in tumor tissue from a majority (73%) of patients diagnosed with CRC, range 14–34% for the different genes. None of these genes was methylated in the 46 paired normal mucosa samples. Interestingly, adenoma tissue also appeared to be methylated in about a third of the patients and the paired normal mucosal tissue unmethylated, although caution is prudent here since we have analysed rather few adenomas.

### Prognosis and methylation of p14^ARF^, p16^INK4a^, APC1A and RASSF1A

When correlating the methylation of the promoter regions of these genes with survival of the CRC patients, we found that methylation of *p14^ARF^* was significantly associated with shorter survival compared to patients that has this gene unmethylated. When adjusting for tumor stage and tumor differentiation this significance was attenuated somewhat. We also saw clear trends towards poorer prognosis when methylation was found in the promoter regions of *RASSF1A* and *APC1A*.

In some of the subjects several of the genes had their promoter regions hypermethylated concurrently, implicating that signalling pathways such as Wnt where *APC1A* are involved, *p16^INK4a^-Rb* and *p14^ARF^-p53*, and normal cell mechanisms such as alkylation and mitotic progression could be altered all at once. Since it has been suggested that CRC evolves from alterations of several of these pathways (26), we thought it relevant to investigate whether this concurrent methylation could affect the outcome for the patient. Grouping together all individuals showing methylation of one or more of the *p14^ARF^*, *RASSF1A* and *APC1A* genes, we obtained an association between promoter hypermethylation and shorter survival which remained statistically significant even after adjusting for tumor stage and differentiation (Fig. 3, Table IV). Thus, promoter hypermethylation of one or more of the genes *p14^ARF^*, *RASSF1A* and *APC1A*, when defined by Pyrosequencing assays, might serve as a marker of poor prognosis which we suggest as a novel, relevant stratification factor in future prospective and interventional studies on CRC.

In other recent studies, poor survival has been reported for patients methylated on the *p14^ARF^* gene (25,27) in agreement with our findings and for *APC1A* one report based on measurements of methylation in plasma DNA reported worse prognosis (28) but another study claimed a better survival in patients with methylated tumor tissue *APC1A*(17). Several reports have claimed predictive value of *p16^INK4a^* methylation (19,21–23,27,29,30) which we could not replicate in our cohort. Data on the prognostic implications of *RASSF1A* methylation are sparse, one study reported a higher prevalence of methylation in liver metastases than in the primary tumor (31).

### Prognosis and methylation of O^6^-MGMT

Promoter methylation in the *O^6^-MGMT* gene has in some studies on glioblastoma been associated with longer survival especially in therapeutic trials using alkylating agents (32–36). It is now proposed that tests for *O^6^-MGMT* methylation status should be included in all future clinical trials in malignant glioma if treatment includes alkylating agents, since it is anticipated that those tests may guide choice of future therapy (37). To asses *O^6^-MGMT* promoter methylation in the mentioned glioma studies, several different methods have been employed, as was recently pointed out by van den Bent *et al*(12), who stressed the point that the CpG island in that part of the gene actually contains almost 100 individual CpG sites and that the used methods only give information on the methylation status of a few of these sites. It has not been clear how many CpG loci, or which ones of them, have to be methylated to achieve *O^6^-MGMT* gene silencing. Our method utilizes a sequence-specific and quantitative assay, Pyrosequencing, to classify the methylation status (13) and thus provides a tool to answer this question. Our assays cover 25 different CpG sites, 18 of which have never been assayed before, and we show here that in the individual patient, the %^Me^C of each of these sites is a characteristic feature of each individual tumor sample, and varies very little from CpG site to CpG site within the same tumor tissue sample (Fig. 1; the same observation holds for *p14^ARF^*, cf. Fig. 2). On the other hand, a considerable inter-individual difference was seen which could be owing to differing degrees of admixture of unmethylated non-neoplastic cells, to true differences in the biology of the tumor, or to a combination of these factors.

There are a few reports on CRC prognosis in relation to methylation of *O^6^-MGMT*, claiming basically no relation (17,29,38). We show here for the first time that like in glioma, promoter methylation of *O^6^-MGMT* in CRC patients tended to confer better long-term survival (Table III), in a context where alkylating agents are rarely an option. This suggests that *O^6^-MGMT* testing might be of a more general interest and warrants to be included not only in planning of glioma treatment but in studies on other malignant neoplasias as well.

### General observations and limitations

A number of methodological differences between the various studies need to be pointed out. Most of the data come from methylation-specific PCR-based methods, not from sequencing-based techniques. Many studies are made on DNA isolated from FFPE samples, which may have decayed due to harsh conditions. We used DNA isolated from fresh tumor tissue, and analysis was performed by bisulphite pyrosequencing (13). The fraction of patients showing methylation of our set of genes ranged from 14% to 34% for the different genes, figures which are both higher (29) and lower (17) than those reported earlier. For instance, one study claiming no prognostic value of *O^6^-MGMT* in CRC classified 60% as methylation-positive by a MS-PCR method (17), as against our figure of 34% using Pyrosequencing which agrees better with the figure of Ogino and coworkers of 38% (38). In future studies, more attention should be payed to the source of DNA and to methods used to classify promoter methylation status, and we contend that Pyrosequencing has earned a place among the methods of choice.

The concept of CIMP-positivity (CpG Island Methylator Phenotype) needs to be relativised based on our findings. The number of subjects simultaneously methylated on 2 or more of the selected set of genes was rather small (Table II) and any predictions based on such a subset of the patient population will therefore have a very limited utility. In contrast, we found a statistically significant negative relation with survival, adjusted for tumor stage and differentiation based on a combined methylation variable defined as having any one or more of the genes *p14^ARF^*, *RASSF1A*, *APC1A* methylated (Table IV, Fig. 3). Employing the same Pyrosequencing assays (13) as in the present paper, we recently showed that *APC1A* promoter methylation was associated with poor prognosis also in cervical cancer (39).

Moreover, methylation of one of the genes in our set, *O^6^-MGMT*, showed a significant association with better survival through the first 60 months following primary surgery. For all these reasons, the concept of a CIMP as a unified set of methylated genes accompanying poor prognosis appears to have limited prospects as a valid prognostic tool in most CRC patients. Perhaps it may ultimately be replaced by a more precise molecular signature associated with poor prognosis (40,41), possibly even tailor-made on the individual basis which might better reflect the random stochastic nature of the processes that characterise sporadic CRC. Such signatures would likely include both chromosomal instability, microsatellite instability, and methylation of a larger set of cancer-related genes than has been currently studied (28,29,30,42,43) as well as less explored features such as histone modifications, nucleosomal occupancy and remodeling, chromatin looping, and non-coding RNAs (44). Hopefully, DNA methylation data may in the future also be useful in guiding adjuvant treatment in CRC as suggested in a recent article (45).

In conclusion, this is the first study to report a significant correlation between CRC patient survival and promoter methylation of the genes *p14^ARF^*, *RASSF1A* and *APC1A*, as defined by Pyrosequencing assay (13), as well as a protective role of *O^6^-MGMT* methylation. Such biomarkers of prognosis in CRC could be utilized as a relevant stratification factor in future prospective and interventional studies on CRC, and might serve as a tool when tailoring treatment for the individual patient. Finally, we maintain that choice of assay methodology may have a determining effect on proper classification of methylation status in the individual patient.

## Figures and Tables

**Table I.** Mean methylated fraction and standard deviation (SD) for all colorectal cancer and adenoma specimens that showed methylation.

Gene	CRC			Adenomas		
	No. (%)	%^Me^C (Mean ± SD)	%^Me^C (Range)	No. (%)	%^Me^C (Mean ± SD)	%^Me^C (Range)
*O^6^-MGMT*	38 (34)	28±11	13–56	3 (30)	24±9	16–33
*p14^ARF^*	32 (29)	36±15	15–90	0 (0)	0±0	-
*p16^INK4a^*	31 (28)	29±10	12–51	1 (10)	22±0	-
*RASSF1A*	16 (14)	31±10	16–61	0 (0)	0±0	-
*APC1A*	30 (27)	38±11	21–67	2 (20)	44±6	41–48

The range of the methylation is also shown here.

**Table II.** Number of colorectal cancer specimens simultaneously methylated on more than one gene.

	*O^6^-MGMT*	*p14^ARF^*	*p16^INK4a^*	*RASSF1A*	*APC1A*
*O^6^-MGMT*	-	15	10	10	12
*p14^ARF^*		-	16	11	7
*p16^INK4a^*			-	7	9
*RASSF1A*				-	5
*APC1A*					-

**Table III.** Multivariate analysis of combined promoter methylation of *0^6^-MGMT*, tumor stage and differentiation in relation to patient survival through 60 months post-surgery.

Variables	No.	Cancer death Rate ratio	95% CI	P-value
Methylation				0.023
No	71	1.00	-	
Yes	35	0.36	0.15–0.87	
Stage				<0.0001
I	17	1.00	-	
II	46	4.35	0.55–34.28	
III	24	8.65	1.08–69.08	
IV	19	40.05	4.98–321.7	
Differentiation				0.151
Well	26	1.00	-	
Moderately	62	0.68	0.29–1.58	
Poorly	18	0.85	0.30–2.40	

**Table IV.** Multivariate analysis of combined promoter methylation of *p14^ARF^*, *RASSF1A* and *APC1A*, tumor stage and differentiation in relation to patient survival.

Variables	No.	Cancer death Rate ratio	95% CI	P-value
Methylation				0.037
No	48	1.00	-	
Yes	58	2.20	1.05–4.62	
Stage				<0.0001
I	18	1.00	-	
II	46	3.82	0.48–30.2	
III	24	7.72	0.96–61.8	
IV	18	31.50	3.99–248.6	
Differentiation				0.330
Well	28	1.00	-	
Moderately	62	0.78	0.34–1.78	
Poorly	16	1.14	0.42–3.10	
